# SWIFT-Review: a text-mining workbench for systematic review

**DOI:** 10.1186/s13643-016-0263-z

**Published:** 2016-05-23

**Authors:** Brian E. Howard, Jason Phillips, Kyle Miller, Arpit Tandon, Deepak Mav, Mihir R. Shah, Stephanie Holmgren, Katherine E. Pelch, Vickie Walker, Andrew A. Rooney, Malcolm Macleod, Ruchir R. Shah, Kristina Thayer

**Affiliations:** SciOme LLC, Research Triangle Park, 2 Davis Drive, 27709 NC, USA; Office of Scientific Information Management, National Institute of Environmental Health Sciences, National Institutes of Health, Department of Health and Human Services, Research Triangle Park, NC USA; Division of the National Toxicology Program, National Institute of Environmental Health Sciences, National Institutes of Health, Department of Health and Human Services, Research Triangle Park, NC USA; Centre for Clinical Brain Sciences, University of Edinburgh, Scotland, UK

**Keywords:** SWIFT-Review, Systematic review, Literature prioritization, Scoping reports, Software

## Abstract

**Background:**

There is growing interest in using machine learning approaches to priority rank studies and reduce human burden in screening literature when conducting systematic reviews. In addition, identifying addressable questions during the problem formulation phase of systematic review can be challenging, especially for topics having a large literature base. Here, we assess the performance of the SWIFT-Review priority ranking algorithm for identifying studies relevant to a given research question. We also explore the use of SWIFT-Review during problem formulation to identify, categorize, and visualize research areas that are data rich/data poor within a large literature corpus.

**Methods:**

Twenty case studies, including 15 public data sets, representing a range of complexity and size, were used to assess the priority ranking performance of SWIFT-Review. For each study, seed sets of manually annotated included and excluded titles and abstracts were used for machine training. The remaining references were then ranked for relevance using an algorithm that considers term frequency and latent Dirichlet allocation (LDA) topic modeling. This ranking was evaluated with respect to (1) the number of studies screened in order to identify 95 % of known relevant studies and (2) the “Work Saved over Sampling” (WSS) performance metric. To assess SWIFT-Review for use in problem formulation, PubMed literature search results for 171 chemicals implicated as EDCs were uploaded into SWIFT-Review (264,588 studies) and categorized based on evidence stream and health outcome. Patterns of search results were surveyed and visualized using a variety of interactive graphics.

**Results:**

Compared with the reported performance of other tools using the same datasets, the SWIFT-Review ranking procedure obtained the highest scores on 11 out of 15 of the public datasets. Overall, these results suggest that using machine learning to triage documents for screening has the potential to save, on average, more than 50 % of the screening effort ordinarily required when using un-ordered document lists. In addition, the tagging and annotation capabilities of SWIFT-Review can be useful during the activities of scoping and problem formulation.

**Conclusions:**

Text-mining and machine learning software such as SWIFT-Review can be valuable tools to reduce the human screening burden and assist in problem formulation.

**Electronic supplementary material:**

The online version of this article (doi:10.1186/s13643-016-0263-z) contains supplementary material, which is available to authorized users.

## Background

In almost every field of scientific inquiry, the current rate of scientific publication is greatly outpacing scientists’ ability to read and assimilate the information. It has been estimated that every year, more than 4000 systematic reviews are conducted and published, each with the goal of summarizing the current state of knowledge relevant to a specific research question [1]. On average, the amount of time required to conduct a single systematic review is at least 6 months to a year [2], and a considerable portion of this time is often spent on formulating the problem and identifying the relevant literature. For this reason, a large number of topics that would benefit from systematic review are waiting in queue and many systematic reviews are out of date by the time they are published.

Various methods taken from the fields of text-mining, machine learning, and information retrieval have the potential to greatly reduce the amount of time it takes to conduct a systematic review and to minimize bias in identifying relevant studies [3, 4]. These methods have much potential to reduce the human burden in screening studies for relevance and to produce “scoping reports” or “scoping studies,” a type of knowledge synthesis undertaken to guide the direction of future research priorities or to help with problem formulation when conducting a systematic review [5]. However, to date, few software systems have been deployed that automate these methodologies, and usage of many of the reported methods requires the assistance of a data scientist having a detailed understanding of statistics and/or the skills necessary to program in statistical programming languages such as R, Python, or Matlab [4]. These factors limit the ability of many systematic review teams to take advantage of these tools and restrict efforts to perform the validation against manual methods that will be required to support routine use.

### Objective and specific aims

Here, we introduce “SWIFT-Review” (SWIFT is an acronym for “Sciome Workbench for Interactive computer-Facilitated Text-mining”), a freely available, interactive workbench that provides numerous tools to assist with problem formulation and literature prioritization. SWIFT-Review can be used to search, categorize, and visualize patterns in literature search results. The software utilizes recently developed statistical modeling and machine learning methods that allow users to identify over-represented topics within the literature corpus and to rank-order titles and abstracts for manual screening.

#### Specific aims

- Describe the methods used by SWIFT-Review to conduct topic modeling, categorization of studies, and priority ranking for relevance.
- Present performance benchmarks for priority ranking based on a comparison of SWIFT-Review to manual review for 20 data sets of various size and complexity. Fifteen of the 20 data sets are public datasets that have been used to evaluate the performance of other text-mining software tools [6].
- Present an example of how SWIFT-Review can be used to prepare a scoping report on an example topic (endocrine-disrupting chemicals; EDCs) selected because of the large size of its literature base and for its complexity in terms of number of chemicals, range of health effects, and types of evidence (human, animal, in vitro).

## Methods

### Document import and search

SWIFT-Review allows users to upload bibliographic records or “documents” (titles and abstracts, plus associated bibliographic data and Medical Subject Heading (MeSH) annotations) either in the form of a file containing a list of PubMed IDs (PMIDs) or the XML resulting from a PubMed search. Although the current version of SWIFT-Review is restricted to data originating from PubMed, the methods described herein are compatible with bibliographic data from other sources. After records have been imported to SWIFT-Review, the application utilizes the Apache Lucene open-source software to provide a search engine and query language which includes Boolean operators, wildcards, and the capability to perform proximity searches to find sets of words occurring near each other in a text, fielded searches to search within specific document sections or by tags, and ranged queries to limit searches on numeric fields to values within a certain range (lucene.apache.org). These features can be used to interactively explore and filter documents using both custom and built-in searches.

### Bag-of-words model to characterize document features

For the purposes of statistical modeling, uploaded documents are represented internally using term frequencies (“bag-of-words” model), where “terms” include both individual words as well as 2- and 3-grams (contiguous sequences of 2 or 3 terms). Separate term counts are maintained for words that occur in a document’s title, abstract, and MeSH headings. For example, if the term “human” occurs in the title, abstract, and as a MeSH heading for a particular document, separate counts are recorded for title:human, abstract:human, and MeSH:human. We also initially considered combining counts for titles and abstracts, but found that this was not helpful (data not shown). All terms in the title and abstract are stemmed using the Porter stemming algorithm [7]; English stop words (a small set of high frequency, low information words including “the,” “of,” “all,” etc.) are removed from the set of individual terms, but not from 2- and 3-gram word phrases.

Raw word counts in each document are converted to length-normalized term frequency-inverse document frequency (TF-IDF) scores [8]. This increases the salience of words that have high frequency in a particular document as compared to the background frequency of that term in the corpus as a whole. For a given document, *d*, let TF_*t,d*_ denote the *term frequency* of term *t* in that document; here, TF_*t,d*_ is simply the raw word count for term *t* in document *d*. The *document frequency* DF_*t*_ is the total number of documents in which TF_*t,d*_ > 0—i.e., the number of documents where term *t* is seen. The *inverse document frequency* is defined as:

$$ {\mathrm{IDF}}_t={ \log}_{10}\frac{N}{{\mathrm{DF}}_t} $$

where *N* is the total number of documents under consideration (in this scenario, the number of documents initially uploaded into SWIFT-Review). Using these components, the TF-IDF_*t,d*_ score is defined as:

$$ \mathrm{T}\mathrm{F}\hbox{-} {\mathrm{IDF}}_{t,d}={\mathrm{TF}}_{t,d}\times {\mathrm{IDF}}_t $$

Hence, words that occur many times in a given document increase its score, but words that occur commonly (i.e., in many different documents) have lower weights.

For notational convenience, each document, *d*, can be represented by a length |*T*| vector, ${X}^d$, where *T* is the set of unique terms found in the full set of documents considered. For a given term, $t\in T$,

$$ {X}_t^d=\mathrm{T}\mathrm{F}\hbox{-} {\mathrm{IDF}}_{t,d} $$

To ensure that documents having many words are not given more importance than documents with fewer words, we normalize ${X}^d$ to have length 1:

$$ {X^d}_{\mathrm{norm}}=\frac{X^d}{\left|{X}^d\right|} $$

For notational simplicity, in the following we will use ${X}^d$ to denote ${X^d}_{\mathrm{norm}}$.

### Topic modeling

Topic modeling is a statistical method used to automatically cluster related documents in a collection of unlabeled texts and to discover computationally derived themes common among those documents. The latent Dirichlet allocation (LDA) topic modeling approach [9, 10] was used to probabilistically assign documents to topics. Under this framework, “topics” are conceptualized as probability distributions over a vocabulary. Given the set of topics for a particular document, each term in the document’s bag of words is assumed to have been generated sequentially by first randomly selecting one of the document’s topics (according to its membership probabilities) and then randomly selecting a word according to that topic’s word distribution. Parameters of this model were estimated using the Mallet LDA package [11]. The result is a set of *K* topics and weighted assignments of each document in the collection to one or more of these topics, where *K* is a parameter that can be set by the user. In the following, the resulting topic membership probabilities are denoted by $Z\left({X}^d\right)$, a length *K* vector whose *i*th element, and $Z{\left({X}^d\right)}_i$ is the probability that document ${X}^d$ originated from the topic *i*.

### Document prioritization

Given a *training set*, which includes examples of manually identified “relevant” and “not relevant” documents in the corpus, SWIFT-Review builds a statistical log-linear model (presented below) to describe the conditional probability that a given document is relevant. This model is then used to estimate the probability that an unlabeled document is relevant. After training the log-linear model, documents are ranked according to their estimated conditional relevance probabilities, $\Pr \left\{Y=1|{X}^d,v\right\}$.

#### Log-linear model

A log-linear model is used for classification. Using the binary variable $Y\in \left\{0,1\right\}$ to denote the relevance (0 = not relevant; 1 = relevant) of document, *d*, our model takes the form

$$ \Pr \left\{Y=y\Big|{X}^d,v\right\}=\frac{e^{v\cdot f\left({X}^d,y\right)}}{e^{v\cdot f\left({X}^d,0\right)}+{e}^{v\cdot f\left({X}^d,1\right)}} $$

In the above notation, $f\left({X}^d,y\right)$ is a vector of real numbers, the *i*th component of which is determined by the *i*th feature function ${f}_i\left({X}^d,y\right),$ which maps a given (document, label) pair to a real number. Under this general framework, feature functions can take a huge variety of forms; here, we use the following two types of features:

1. Word score features: ${f}_i\left({X}^d,y\right)={X}_i^d$ (i.e., the normalized TF-IDF score for term *i*).
2. Topic weight features: ${f}_{\left|T\right|+i}\left({X}^d,y\right)=Z{\left({X}^d\right)}_i$ (i.e., the probability the document belongs to topic $i\in \left\{1,..,K\right\}$.

Hence, $f\left({X}^d,y\right)$, is a length |*T*| + *K* vector of real numbers.

The weight vector, *v*, is used to quantify the “strength” of each feature in determining the relationship between features and the conditional probability. Weights are estimated by maximum likelihood using the labeled training data. Under the above model, the (log) likelihood function over *n* training documents is as follows:

$$ L(v)={\displaystyle \sum_{d=1}^n}v\cdot f\left({X}^d,{Y}^d\right)-{\displaystyle \sum_{d=1}^n} \log {\displaystyle \sum_{y\in \left\{0,1\right\}}}{e}^{v\cdot f\left({X}^d,y\right)} $$

In order to avoid over-fitting the parameter vector, we include a regularization penalty, $\frac{1}{2}\lambda \sum_{i=1}^{\left|T\right|+K}{v}_i^2$, where *λ* is a parameter that controls the size of the penalty. With this modification, the regularized likelihood function becomes

$$ L(v)={\displaystyle \sum_{d=1}^n}v\cdot f\left({X}^d,{Y}^d\right)-\left({\displaystyle \sum_{d=1}^n} \log {\displaystyle \sum_{y\in \left\{0,1\right\}}}{e}^{v\cdot f\left({X}^d,y\right)}\right)-\frac{1}{2}\lambda {\displaystyle \sum_{i=1}^{\left|T\right|+K}}{v}_i^2 $$

The likelihood equation is maximized using the limited memory Broyden-Fletcher-Goldfarb-Shanno (LBFGS) algorithm, a gradient-based optimization procedure designed for high-dimensional parameter spaces [12].

### Assessing document prioritization performance

#### Datasets

Prioritization methods were tested on 20 datasets that were previously curated manually by reviewers (Table 1). Data sets were selected to allow comparison with other text-mining software and represent a range of size (~300 to ~49,000) and complexity, including both focused literature topics and broad literature topics. Four datasets (Additional file 1) were generated by the National Toxicology Program (NTP) Office of Health Assessment and Translation (OHAT), one dataset (Additional file 2) was provided by the Edinburgh CAMARADES group (www.camarades.info), and the remaining 15 datasets are public data sets that have been used to assess the performance of other priority-ranking methods [6]. Eighteen of the 20 data sets used PubMed records (titles, abstracts, and MeSH terms) as the input and 2 used titles and abstracts identified from a search of multiple databases.

**Table 1:** Summary of datasets used to assess priority ranking performance

Data set	Source	Database (inputs)	Records from search	Included	Excluded	Comments
PFOA/PFOS and immunotoxicity	NIEHS	PubMed (PMIDs)	6331	95 (1.5 %)	6236 (98.5 %)	Targeted topic^a^
Bisphenol A (BPA) and obesity	NIEHS	PubMed (PMIDs)	7700	111 (1.4 %)	7589 (98.6 %)	Targeted topic
Transgenerational inheritance of health effects	NIEHS	PubMed (PMIDs)	48,638	765 (1.6 %)	47,873 (98.4 %)	Untargeted topic
Fluoride and neurotoxicity in animal models	NIEHS	Multiple (titles + abstracts)	4479	51 (1.1 %)	4428 (98.9 %)	Targeted topic
Neuropathic pain	CAMARADES	Multiple (titles + abstracts)	29,207	5011 (17.2 %)	24,196 (82.8 %)	Semi-targeted topic
Skeletal muscle relaxants	[6]	PubMed (PMIDs)	1643	9 (0.6 %)	1634 (99.4 %)	Public dataset
Opioids	[6]	PubMed (PMIDs)	1915	15 (0.8 %)	1900 (99.2 %)	Public dataset
Antihistamines	[6]	PubMed (PMIDs)	310	16 (5.2 %)	294 (94.8 %)	Public dataset
ADHD	[6]	PubMed (PMIDs)	851	20 (2.4 %)	831 (97.6 %)	Public dataset
Triptans	[6]	PubMed (PMIDs)	671	24 (3.6 %)	647 (96.4 %)	Public dataset
Urinary Incontinence	[6]	PubMed (PMIDs)	327	40 (12.2 %)	287 (87.8 %)	Public dataset
Ace Inhibitors	[6]	PubMed (PMIDs)	2544	41 (1.6 %)	2503 (98.4 %)	Public dataset
Nonsteroidal anti-inflammatory	[6]	PubMed (PMIDs)	393	41 (10.4 %)	352 (89.6 %)	Public dataset
Beta blockers	[6]	PubMed (PMIDs)	2072	42 (2.0 %)	2030 (98.0 %)	Public dataset
Proton pump inhibitors	[6]	PubMed (PMIDs)	1333	51 (3.8 %)	1282 (96.2 %)	Public dataset
Estrogens	[6]	PubMed (PMIDs)	368	80 (21.7 %)	288 (78.3 %)	Public dataset
Statins	[6]	PubMed (PMIDs)	3465	85 (2.5 %)	3380 (97.5 %)	Public dataset
Calcium-channel blockers	[6]	PubMed (PMIDs)	1218	100 (8.2 %)	1118 (91.8 %)	Public dataset
Oral hypoglycemics	[6]	PubMed (PMIDs)	503	136 (27.0 %)	367 (73.0 %)	Public dataset
Atypical antipsychotics	[6]	PubMed (PMIDs)	1120	146 (13.0 %)	974 (87.0 %)	Public dataset

^a^Targeted topics refers to examples where a specific exposure and health outcome were identified (e.g., bisphenol A and obesity); for untargeted topics, one or both of these parameters were not defined, e.g., the topic transgenerational inheritance of health effect focused on a particular study design and was not restricted to a specific type of exposure or health outcome

#### Performance metrics

The “Work Saved over Sampling” (WSS) performance metric [6] and percentage of documents screened were used to evaluate the prioritization procedure described above. The WSS defines, for a specific level of recall, the percent reduction in effort achieved by a ranking method as compared to a random ordering of the documents. Specifically,

$$ \mathrm{W}\mathrm{S}\mathrm{S}@R=\frac{\mathrm{TN} + \mathrm{F}\mathrm{N}}{N}-\left(1.0-R\right) $$

where TN denotes true negatives, FN denotes false negatives, *N* denotes the total size of the data set, and *R* is the desired level of recall ($R=\frac{\mathrm{TP}}{\mathrm{TP}+\mathrm{F}\mathrm{N}}$, with TP denoting true positives). For example, $\mathrm{W}\mathrm{S}\mathrm{S}@.95=\frac{\mathrm{TN}+\mathrm{F}\mathrm{N}}{N}-.05$.

**Fig. 1:**
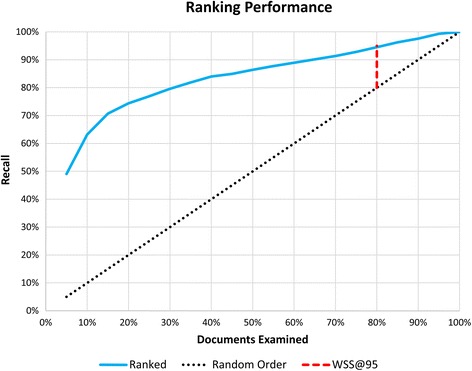
“Work Saved over Sampling” (WSS) performance metric. The *dotted black line* illustrates the expected recall achieved when traversing a randomly ordered list. Similarly, the *blue line* shows the recall obtained when traversing a (hypothetical) ranked list. The length of the *dotted red line* indicates the percent reduction in effort achieved by ranking and corresponds to the WSS at 95 % recall, in this case, approximately 15 % (95–80 %)

The maximum possible WSS score is 1, indicating a 100 % reduction in screening burden. A WSS score of 0 or less indicates that random ordering would be just as effective or more effective than priority ranking. In a plot of recall as a function of the number of ranked documents screened, the WSS at a specific level of recall is simply the distance from a straight line with slope = 1 (Fig. 1).

The percentage of documents screened (to obtain the desired recall) is related to WSS@*R* as follows:

$$ \begin{array}{c}\mathrm{P}\mathrm{ercentage}\ \mathrm{screened}=\frac{\mathrm{TP}+\mathrm{F}\mathrm{P}}{N}\\ {}=1-\frac{\mathrm{TN}+\mathrm{F}\mathrm{N}}{N}\\ {}=R-\mathrm{W}\mathrm{S}\mathrm{S}@R\end{array} $$

#### Test procedure

To make our results comparable with other published results obtained using the Cohen benchmark datasets [6], we applied the following testing procedure. First, after random shuffling, each dataset was divided such that half of the entire dataset was used for training and the remainder for testing. Similar to Cohen (2008/2011), we used stratified sampling to ensure that the test and training sets had the same percentage of relevant documents. The following algorithmic parameters were chosen by cross-validation on the training sets: *K*, *λ*, inclusion/exclusion of MeSH terms, and inclusion/exclusion of 2- and 3-grams. For each dataset, the WSS@95 was computed using the test set and averaged over 25 trials.

### Document tagging for problem formulation

**Fig. 2:**
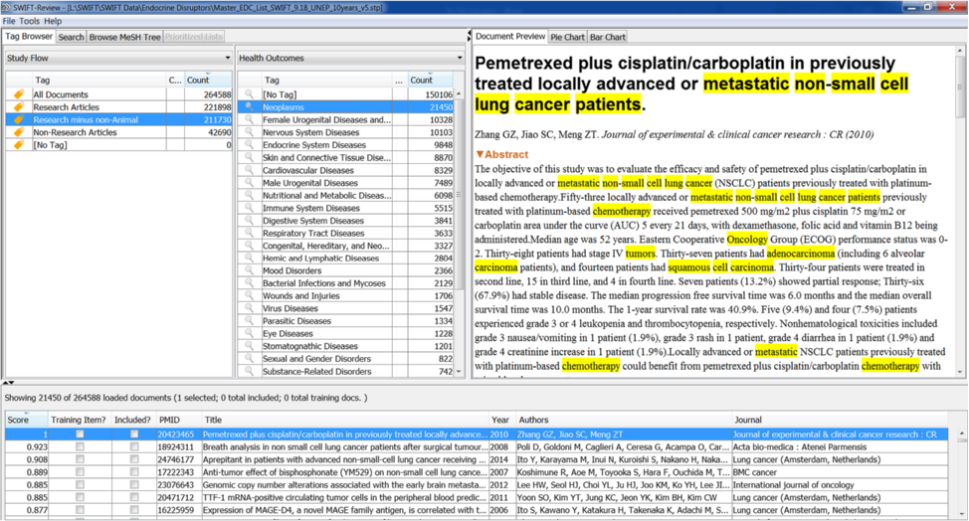
SWIFT-Review user interface and tag browser. The SWIFT-Review Tag Browser allows users to interactively filter a literature set by selecting various combinations of “tags” that have been automatically and/or manually applied to the corpus. In this case, the user has selected for investigation research articles in the “Neoplasms” health outcome category; terms in each abstract that are related to these tags are highlighted automatically in the *Document Preview* panel

When documents are loaded into SWIFT-Review, each record is automatically associated with various “tags” which are used to label documents according to meaningful categories. Users can then interactively filter the imported documents according to these tags by using the SWIFT-Review “Tag Browser” (Figs. 2 and 7). SWIFT-Review tags may include various imported meta-data such as MeSH Terms and MeSH Supplementary Concept Records, various entities or topics automatically extracted from documents, and any other label applied manually by the user. In addition, SWIFT-Review includes several built-in Lucene search filters that can be used to automatically tag documents such as health outcome, evidence stream (human, animal, in vitro), or chemical treatment. These search filters, which are described below, are included in SWIFT-Review by default because the initial development work was done to address literature-mining needs in environmental and occupational health, but users can also integrate their own custom search strategies which can be used to tag documents according to the specific requirements for a given project.

#### Evidence stream

A customized search filter was developed to identify and tag human studies with no restriction on study design (i.e., randomized clinical trial or case report would both be identified.) A search strategy to identify animal models was created by modifying a strategy for identifying animal research from [13]. In brief, the modifications entailed consolidating the search term list to focus on animal models most relevant to environmental health studies by removing those animals of less interest; e.g., bison, panda, sable. Also, the Hooijmans et al. strategy only searches for animal terms in the title and abstract fields if the PubMed record has not been indexed with MeSH. The SWIFT-Review search strategy does not have that limitation. The query used to identify and tag in vitro studies was based on MeSH headings as well as text words for specific cell lines. The search strategies described above for human, animal, and in vitro studies are available in Additional file 3.

#### Health outcomes

The top level PubMed MeSH disease codes (C01, C02, C03, …, C26) and mental disorder codes (F03.075, F03.080, F03.087, …, F03.900) are used to tag health outcomes in the imported documents. In addition, in order to automatically highlight relevant words and phrases in these tagged documents within the SWIFT-Review user interface (Fig. 2), we have also employed a semi-automated approach to “mine” PubMed and find relevant terms that are enriched for each of these MeSH codes. For each top level MeSH code, we randomly selected 5,000 documents from PubMed that were previously labeled with that code by National Center for Biotechnology Information (NCBI) annotators. After computing the ${\overline{X}}_{\mathrm{norm}}$, averaged over the selected documents for each top level code, we then sorted the resulting term lists by their normalized TF-IDF scores and retained the top 500 most salient terms for each category. Finally, each list was manually reviewed to remove high scoring terms that were deemed to still lack specificity. For example, in the case of MeSH code C08 “Respiratory Tract Diseases”, we removed the terms “title:obstruct,” “title:cancer,” and “abstract:niv.” Table 2 contains the top 25 terms in the list of 456 terms selected to represent MeSH code C08. The resulting set of keywords is used by SWIFT-Review to automatically highlight terms associated with each health outcome (Fig. 2).

**Table 2:** The 25 top-scoring terms for MeSH code C08 “Respiratory Tract Diseases”

Word	Type	TF	DF	TF_IDF score
Pulmonari	Title	708	1777	0.372008
Lung	Title	715	1623	0.362747
Lung neoplasms	MESH	746	2003	0.269274
Lung	Abstract	2459	3131	0.266611
Tuberculosis, pulmonary	MESH	324	940	0.211322
Lung cancer	Title 2-gram	241	474	0.209402
Pulmonari	Abstract	1564	2571	0.204265
Asthma	Title	281	889	0.1953
Asthma	MESH	486	1675	0.193189
Respiratori	Title	292	1024	0.175736
Lung diseases	MESH	304	910	0.16359
Asthma	Abstract	1045	1163	0.158508
Tuberculosi	Title	233	1053	0.153724
Lung cancer	Abstract 2-gram	588	689	0.145245
Pneumonia	Title	178	547	0.139709
Bronchial	Title	139	327	0.13054
Pulmonari tuberculosi	Title 2-gram	86	236	0.112054
Small cell lung	Title 3-gram	92	174	0.110415
Cell lung cancer	Title 3-gram	87	161	0.10728
Pulmonari diseas	Title 2-gram	74	146	0.105468
Pulmonari hypertens	Title 2-gram	67	118	0.100461
Chronic obstruct	Title 2-gram	71	124	0.098575
Chronic obstruct pulmonari	Title 3-gram	56	99	0.095042
Obstruct pulmonari diseas	Title 3-gram	55	99	0.093811
Pulmonari embol	Title 2-gram	52	94	0.090121

#### Chemical exposure or treatment

##### Tox21 chemicals

Toxicology Testing in the 21st Century (Tox21) is a pooling of US federal resources and expertise from the National Institutes of Environmental Health Sciences/National Toxicology Program (NIEHS/NTP), US Environmental Protection Agency (EPA), National Institutes of Health/National Center for Advancing Translational Sciences (NIH/NCATS), and the US Food and Drug Administration (FDA) to use robotics technology to screen thousands of chemicals for potential toxicity, use screening data to predict the potential toxicity of chemicals, and develop cost-effective approaches for prioritizing the thousands of chemicals that need toxicity testing [14]. Currently, 8186 unique chemicals are being screened, including a diverse set of environmental chemicals, pharmaceuticals, and endogenous compounds. Using the complete list of unique Tox21 chemicals downloaded from the EPA website [15], SWIFT-Review automatically scans the title, abstract and MeSH headings for each document to find occurrences of these chemicals within documents. Literature search strategies for identifying and tagging Tox21 chemicals were automatically constructed by using (1) the common name for the chemical as presented in the source reports listed above, (2) the Chemical Abstract Services Registry Number (CASRN), and (3) and retrieval of synonyms from the ChemIDPlus database which currently contains chemical names and synonyms for over 400,000 chemicals [16]. In total, there are more than 2.7 million names in the ChemIDPlus database; however, many of these synonyms are ambiguous and could lead to false positives. Most of these ambiguous terms are (1) short alphanumeric sequences that could be confused with arbitrary acronyms or abbreviations (e.g., “2VP” for “2-vinylpyridine”), (2) English words that have been used as industrial trade names, street drug slang, etc., or (3) chemical formulas that do not unambiguously define a chemical. Hence, to avoid false positives, the list was filtered as follows:

- Excluded all names of type “DisplayFormula” (i.e., chemical formulas like “H20”).
- Obtained a set of 109,582 English words from SIL International Linguistics [17]. Any chemical terms that appeared in this list and were not the exact name of a Tox21 chemical (i.e., a synonym and not the original name) were removed. This removed ambiguous terms like “stuff” and “impact” but not “ethanol” or “toluene.”
- Removed all terms with fewer than five letters (most of the ambiguous abbreviations).
- Removed non-English chemical names.
- Removed inverted chemical names.

On average, the Tox21 chemicals have a mean of 20 synonyms and a median of 16 synonyms. The full list of Tox21 names and synonyms (156,304 terms) is available in Additional file 4.

To identify the literature relevant to endocrine disrupting chemicals, the resulting sets of chemical synonyms were also used to create PubMed queries of the form: “CHEMICAL_NAME”[tiab] OR “CASRN”[rn] OR “S1” OR “S2” … OR “Sn” where “CHEMICAL_NAME” is the original chemical name, “CASRN” is the corresponding CAS number, and S1 through Sn are the synonyms from ChemIDPlus. When the chemical name had an exact match to a MeSH term or supplementary concept, we also included those terms in conjunction with the PubMed [mh_noexp] and/or [supplementary concept] fields. In order to make the published queries more readable, we used PubMed’s “search details” and “quoted phrase not found” features, which provide details about which query terms are not found in the database, to eliminate synonyms that resulted in no hits from PubMed.

##### Broad categories of exposure

Targeted literature search strategies were manually developed to allow SWIFT-Review to tag (Additional file 5) documents under the following broad categories of exposure: air pollution, allergens, diet and nutrition, endocrine disruptors, flame retardants, heavy metals, ionizing radiation, miscellaneous, occupational, pesticides, phthalates, polycyclic aromatic hydrocarbons, solvents, stress, and general environmental exposures.

#### Dataset used to assess document tagging and annotation features: Endocrine-disrupting chemicals

##### Specific chemicals used to establish literature corpus

SWIFT-Review document tagging and annotation were used to assess the extent and nature of the literature during the last 10 years for 171 chemicals implicated as endocrine disruptors in the 2012 World Health Organization (WHO)/United Nations Environment Programme (UNEP) report “The State-of-the-Science of Endocrine Disrupting Chemicals” [18]. Endocrine disrupting chemicals are substances that may mimic or interfere with the function of hormones in the body. As a result, EDCs may turn on, shut off, or modify signals that hormones carry, which can affect the normal functions of a broad range of tissues and organs [19]. Many of these substances have been linked with developmental, reproductive, neural, immune, and other problems in humans, wildlife, and laboratory animals.

In brief, literature search strategies for the 171 chemicals were automatically constructed using the approach described above (“Tox21 chemicals”) and the automated search results were manually proof-read to remove other terminology likely to result in retrieval of irrelevant documents. Search strategies for each chemical are presented in Additional file 6. The searches were run in PubMed and the results were uploaded into the software, which automatically applied the tagging procedures described above. Using SWIFT-Review’s interactive tag browser, we further refined the literature corpus, using the MeSH publication type filter to identify and eliminate non-research articles and the SWIFT evidence stream filter to identify and eliminate plant studies. The remaining documents were categorized and visualized according to health outcome, evidence stream, and chemical name.

## Results

### Performance of prioritization algorithm

**Fig. 3:**
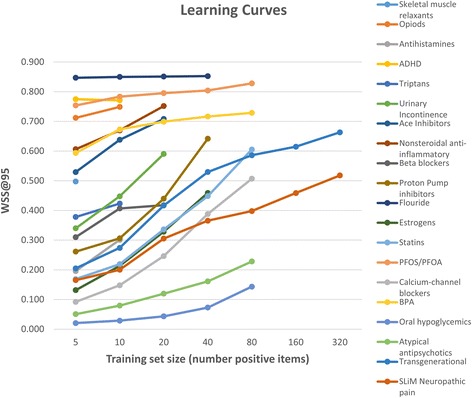
Learning curves. The graphs above show that, as expected, performance of the prioritization method on each dataset is an increasing function of training set size. Since the total number of available positive instances varies significantly between datasets, not all sizes could be tested for each dataset

**Fig. 4:**
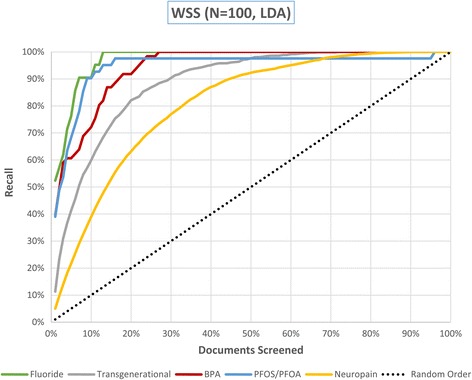
Performance of ranking algorithm on five datasets: Transgenerational, BPA, PFOS/PFOA, Neuropain: *N* = 100 [50 included; 50 excluded.]; Fluoride: *N* = 60 [30 Included; 30 Excluded.]) In all cases, the ranking algorithm results in a substantial potential reduction in screening effort compared to random ordering, with WSS@95 scores ranging from about 60 % (neuropain) to 90 % (fluoride)

We report the WSS@95 scores obtained for each of the 20 datasets in Table 3. Compared with the reported performance of other tools using the same datasets, the SWIFT-Review ranking procedure obtained the highest scores on 11 out of 15 of the public datasets [6]. Cohen’s SVM classifier [6] achieved the highest scores on the remaining four datasets. In general, the priority ranking performance was better for the datasets from NIEHS and CAMARADES; the mean WSS@95 score was 48.8 % for the 15 previously published datasets and 76.6 % for the 5 new datasets introduced here. Figure 3 shows how the performance on each dataset changes as a function of the number of training items (assuming a balanced training set with equal number of positive and negative instances). In all cases, as expected, performance appears to be an increasing function of training set size. In addition, Fig. 4 shows the recall achieved on the 5 new datasets as a function of the total documents screened, after training the algorithm with a seed size of 50 included and 50 excluded documents.

**Table 3:** Summary of SWIFT performance ranking metrics

	Cohen (2006) [6]	Matwin (2010) [28]	Cohen (2008/11) [29, 30]	SWIFT-Review (25 trials)
	WSS@95 [proportion of studies screened to achieve 95 % recall]			
PFOA/PFOS and immunotoxicity	N/A	N/A	N/A	0.805 [0.145]
Bisphenol A (BPA) and obesity	N/A	N/A	N/A	0.752 [0.198]
Transgenerational inheritance of health effects	N/A	N/A	N/A	0.714 [0.236]
Fluoride and neurotoxicity in animal models	N/A	N/A	N/A	0.870 [0.080]
Neuropathic pain	N/A	N/A	N/A	0.691 [0.259]
SWIFT-Review mean				0.766 [0.184]
Skeletal muscle relaxants	0.000 [0.950]	0.265 [0.685]	0.374 [0.576]	**0.556 [0.394]**
Opioids	0.133 [0.817]	0.554 [0.396]	0.364 [0.586]	**0.826 [0.124]**
Antihistamines	0.000 [0.950]	0.149 [0.801]	**0.236 [0.714]**	0.137 [0.813]
ADHD	0.680 [0.270]	0.622 [0.328]	0.526 [0.424]	**0.793 [0.157]**
Triptans	0.034 [0.916]	0.274 [0.676]	0.346 [0.604]	**0.412 [0.538]**
Urinary incontinence	0.261 [0.689]	0.296 [0.654]	0.432 [0.518]	**0.530 [0.420]**
Ace inhibitors	0.566 [0.384]	0.523 [0.427]	0.733 [0.217]	**0.801 [0.149]**
Nonsteroidal anti-inflammatory	0.497 [0.453]	0.528 [0.422]	0.672 [0.278]	**0.730 [0.220]**
Beta blockers	0.284 [0.666]	0.367 [0.583]	**0.465 [0.485]**	0.428 [0.522]
Proton pump inhibitors	0.277 [0.773]	0.229 [0.721]	0.328 [0.622]	**0.378 [0.572]**
Estrogens	0.183 [0.767]	0.375 [0.575]	0.414 [0.536]	**0.471 [0.479]**
Statins	0.247 [0.803]	0.315 [0.635]	**0.491 [0.459]**	0.436 [0.514]
Calcium-channel blockers	0.122 [0.828]	0.234 [0.716]	0.430 [0.520]	**0.448 [0.502]**
Oral hypoglycemics	0.090 [0.860]	0.085 [0.865]	**0.136 [0.814]**	0.117 [0.833]
Atypical antipsychotics	0.141 [0.809]	0.206 [0.744]	0.170 [0.780]	**0.251 [0.699]**
Mean (Cohen benchmark)	0.234 [0.716]	0.335 [0.615]	0.408 [0.542]	**0.488 [0.462]**
SWIFT-Review grand mean				0.540 [0.410]

Metrics shown are the WSS@95 and (in brackets) the proportion of studies screened to achieve 95 % recall. When applicable, bold text indicates the method with the highest performance (highest WSS) for each dataset

**Fig. 5:**
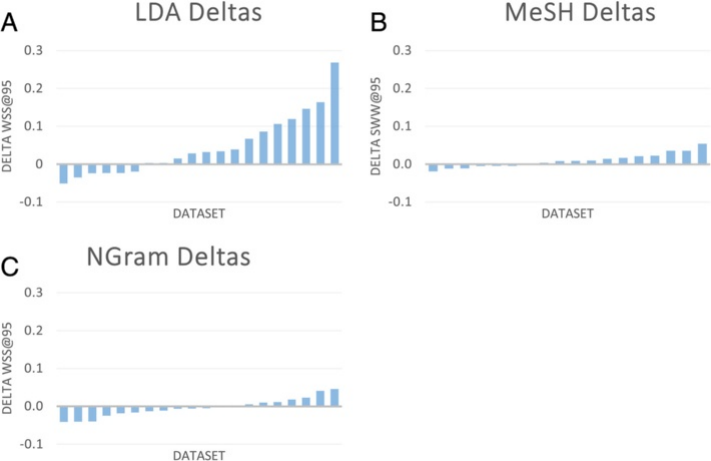
Observed changes in WSS@95 attributable to three feature types. **a** LDA, **b** MeSH terms, and **c** N-grams on 20 SR datasets. Mean changes in WSS@95 were 4.4 % (LDA), 1 % (MeSH), and −0. 4 % (NGrams). In each case, performance was measured on each of the 20 datasets both with and without the specified feature type. The resulting WSS@95 differences for each dataset were averaged over 25 trials. As shown in **a**, adding LDA features to the ranking algorithm can result in significant performance increases, whereas inclusion of the MeSH and NGram features (**b** and **c**) were not found to result in large additional benefits when the remaining feature types were also included

We also assessed the effects of three major feature types: MeSH terms, N-Grams, and topic model membership (Fig. 5). The estimates were obtained by observing the effect of systematically removing those feature types and comparing the results to the original results obtained using all features. As shown in Fig. 5, availability of MeSH annotations is not critical for success of the procedure. In fact, inclusion of MeSH annotations only improved performance by an increase of 1 % WSS@95 (on average) and actually harmed the performance for some individual tests. Similarly, the overall effect of including *n*-grams was also negligible when the other features were available. Topic modeling, on the other hand, provided an average increase of about 4.4 % WSS@95. To characterize the features and feature types that contributed maximally to each classifier, we surveyed the most highly weighted features for the four NIEHS datasets (Additional file 7). In most cases, the highly weighted features appear to be sensible. For example, the features with the most highly negative weights (i.e., most indicative of the excluded class) for the bisphenol A (BPA) obesity dataset include several topics related to dental procedures. Many of the documents in this dataset were retrieved because BPA is commonly used in dental sealants, but these were excluded as not being relevant to the research question studied.

### EDC case study: use of SWIFT-Review document tagging and annotation

**Fig. 6:**
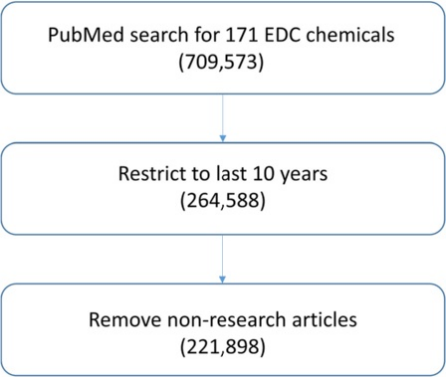
Study Flow diagram for the analysis of 171 UNEP EDC chemicals. The literature search identified 221,898 recent research articles out of the total 709,573 EDC articles retrieved from searching PubMed

We utilized SWIFT-Review’s document tagging and annotation capabilities to perform a scoping exercise on a set of EDC chemicals; the study flow diagram for the analysis of 171 UNEP EDC chemicals is displayed in Fig. 6. The initial PubMed search yielded 709,573 hits in total. By limiting the search to PubMed results from the last 10 years, the literature corpus was reduced to 264,588 records. This allowed us to focus on recent research trends. These citations were uploaded into SWIFT-Review, which was then used to filter out non-research articles (e.g., reviews or commentaries), reducing the size of the corpus to 221,898 documents (Fig. 6).

**Fig. 7:**
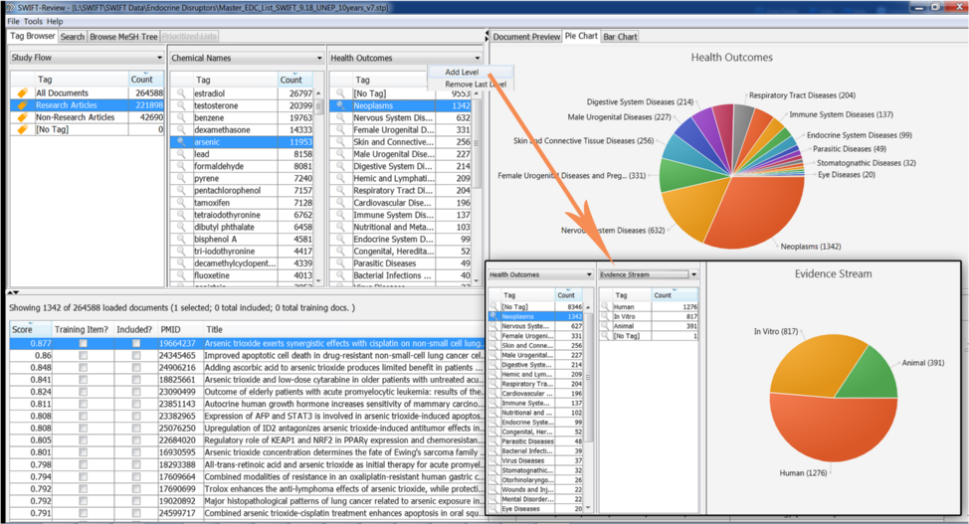
Interactively exploring arsenic in the EDC scoping report in SWIFT-Review. This example shows a pie chart survey of health outcomes represented among the 2400 studies on arsenic with a MeSH disease code. In this pie chart, studies lacking a MeSH disease code are not displayed (9553 of the 11,953 documents retrieved for arsenic) and documents may appear in multiple health outcome categories. Below the pie-chart is a list of 1342 documents relevant to “arsenic and neoplasms”. *Inset* Using the interactive browser, users can “drill down” to further explore documents in a specific area, e.g., arsenic and neoplasms, based on other tags such as evidence stream (human, animal, in vitro)

**Fig. 8:**
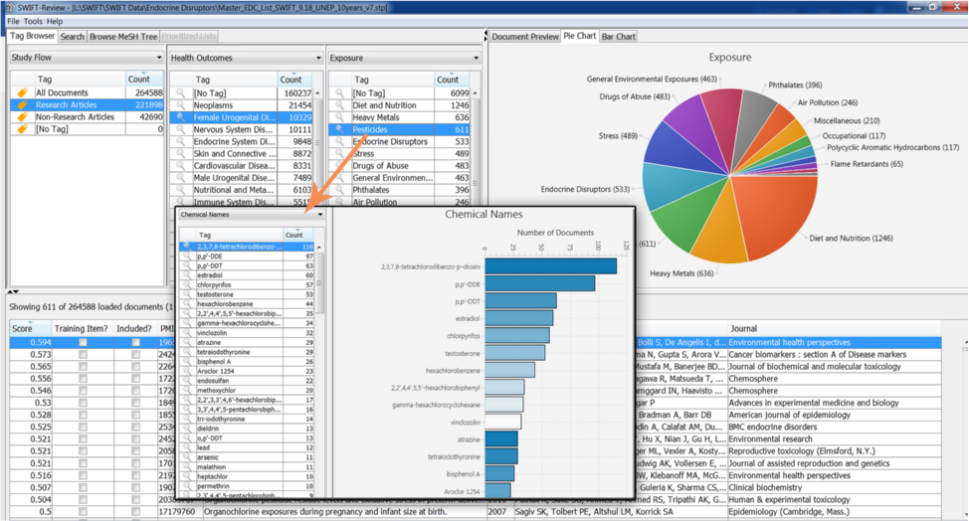
Survey of types of chemicals associated with female urogenital disease and pregnancy. The current example uses a pie chart graphic to survey the types of stressors (e.g., pesticides, drugs of abuse, diet and nutrition) associated with the health outcome of female urogenital disease and pregnancy. Below the pie chart is a list of 611 documents retrieved as part of the “pesticides” filter within SWIFT-Review and a bar chart of the most common Tox21 chemicals referenced in the pesticides cluster. Note that bisphenol A is not a pesticide but appears on this list because it was frequently mentioned in the pesticide studies

During the import, SWIFT-Review automatically annotated the uploaded documents using tags relevant to the environmental health sciences, including chemical exposure, evidence stream (human, animal, in vitro), and health outcome. These tags can then be used to interactively explore and “drill-down” to investigate specific aspects of the literature corpus (Figs. 7 and 8), moving from a visualization of bodies of evidence by chemical, or health outcome to the actual studies reporting data. The document prioritization capabilities of SWIFT-Review can then be applied to specific areas of interest in the corpus (e.g., association of a particular EDC with a specific health outcome such as arsenic and neoplasms) providing users with strategies to conduct survey-level analyses of a topic and identify the number of potentially relevant studies for subsequent systematic review. The list of studies supporting each health outcome or evidence stream can be rapidly accessed by clicking on the interactive figure, and users can pull up the abstract and full study details for individual studies within the areas of interest.

**Fig. 9:**
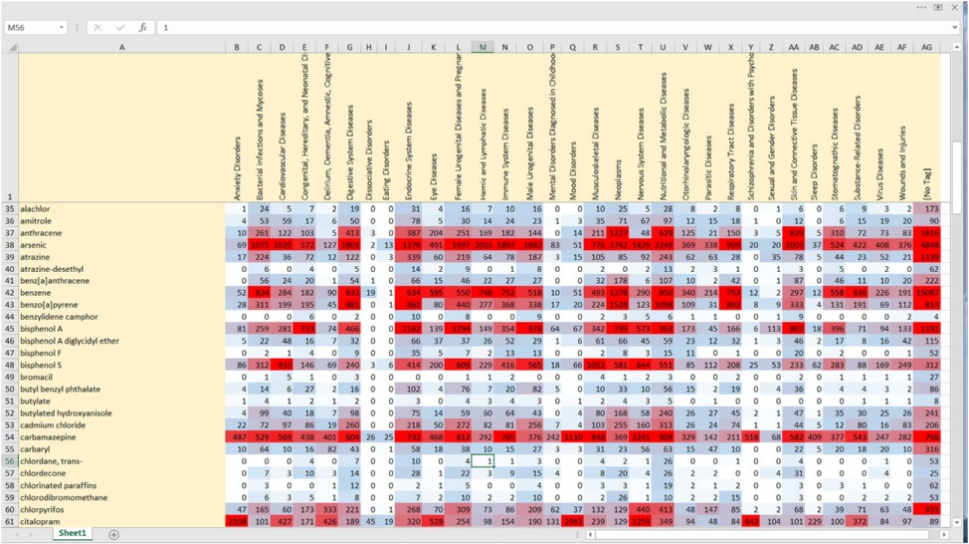
Excerpt of a heat map displaying search results for the 171 EDC chemicals categorized by health outcomes. The numbers displayed indicate the number of SWIFT-Review records matching each combination of chemical (*rows*) and health outcomes (*columns*). “Pockets” with larger numbers of matching records are displayed in red color

**Fig. 10:**
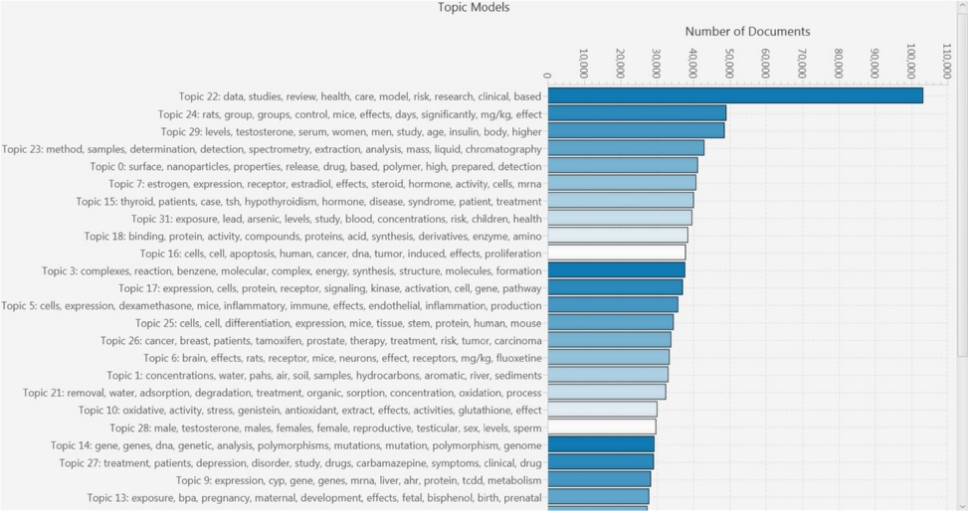
Topic models bar chart from the EDC scoping report. Topic modeling is an unsupervised clustering technique that can often automatically “discover” the main themes in an unlabeled literature corpus. For example, in the case of the EDC literature set, several interesting topics are shown above including topics related to BPA exposure during pregnancy (topic 13), analytical methods used to measure levels of EDCs (topic 23), estrogen, expression, and receptors (topic 7), lead and arsenic exposure (topic 31), breast and prostate cancer (topic 26), and thyroid disease (topic 15). Within SWIFT-Review, users can select any of these topics to interactively browse the associated documents

In addition, various visualizations (e.g., Figs. 9 and 10) are provided which can be helpful during the processes of scoping and problem formulation in which one seeks to assess the current state of the science, identify questions for which the literature base is data rich or data poor, and discover interesting “pockets” of literature relevant to a particular research topic. LDA topic modeling can also be used during these endeavors to automatically identify potentially important “themes” in a bibliographic corpus because those themes are over-represented and to browse the documents within those topics. For example, Fig. 10 shows several of the subjects automatically identified in the EDC dataset. As shown in the figure, topics can be characterized using the set of automatically identified words with the highest conditional probability; in most cases, after cursory examination, these topics can then be assigned meaningful short names. For example, the topics shown here include analytical methods used to measure levels of EDCs (e.g., topic 23), measures of exposure (e.g., topic 31 on lead and arsenic or topic 1 on polycyclic aromatic hydrocarbons (PAHs)), and health outcome topic groupings (e.g., topic 26—breast and prostate cancer and topic 15—thyroid disease).

## Discussion

### Document prioritization

Here, we have tested automated document prioritization on 20 previously conducted systematic review datasets, and the results presented clearly suggest that using machine learning to triage documents for screening has the potential to save, on average, more than 50 % of the screening effort ordinarily required when using un-ordered document lists (Table 3). To the best of our knowledge, the performance benchmarking of SWIFT-Review for use in the screening phase of systematic review reported here is the most extensive conducted to date. Compared to other algorithms previously tested on 15 of the datasets, our procedure performs favorably and obtains the best WSS@95 scores on the majority of the datasets (11 out of 15). We have also introduced five new systematic review datasets (Additional files 1 and 2), which can be used, in the future, to benchmark further developments in the field. Compared to other datasets available for benchmarking, these five new datasets are much larger (range from 4479 to 48,638 studies) and more complex with respect to the type of study considered relevant (e.g., relevance is based on a specific study design for transgenerational and evidence from human, animal, and in vitro studies were considered relevant for BPA and PFOS/PFOA). One fundamental difference between the various datasets may be the search procedure used to obtain the initial corpus. For example, compared to some of the other datasets, the NIEHS literature searches may place more emphasis on recall over precision, potentially leading to more “low hanging fruit” for the classification algorithm to eliminate. Similarly, the observed performance on the transgenerational data was worse than performance on the more targeted topics such as the PFOS/PFOA and BPA datasets because of the lack of consistency in defining the concept of translational inheritance in the literature and the broad range of literature considered relevant, i.e., relevance was based on utilization of a certain study design with no restriction on type of exposure or health outcome (see protocol for the transgenerational inheritance systematic review for more detail [20]).

Although most of the data sets (18 of 20) used in the current analysis are based on PubMed searches, the ranking methodology available in SWIFT-Review is applicable to any set of scientific titles and abstracts, including those derived from non-PubMed bibliographic sources. The inclusion of PubMed-specific MeSH terms was found to result in only a minor improvement to ranking performance. In fact, in this study, the data set with the highest WSS@95 score was fluoride, one of the two datasets for which MeSH terms were unavailable.

In a recent related work [21], it was noted that LDA topic modeling resulted in performance inferior to simple alphanumeric features on a similar classification task. However, in that instance, topic models were used *instead of* alphanumeric features; here, we use topic models *in addition to* the bag of words model. We observed that adding LDA features increased the overall performance by an average of +4.4 % WSS@95. The two datasets that benefitted most from LDA were the two datasets with the smallest number of positive instances available for training: skeletal muscle relaxants and opioids. For these two datasets, adding LDA features increased the performance by +16.4 % and +26.8 % WSS@95, respectively. Since LDA is an unsupervised algorithm, it is expected that it may confer the largest benefit when the total number of labeled documents is small, but when many unlabeled documents are available. For these reasons, the prioritization module available in SWIFT-Review automatically computes and includes these features.

It should be noted that the testing procedure used to compare ranking performance to previous work assumes that one half of the total available data is available for training. While this may be obtainable in practice for scenarios where the task is to update an existing review, for new reviews, the number of seed documents will most likely be much smaller. As shown in Fig. 3, ranking performance is an increasing function of training set size. On the other hand, we have observed excellent performance using our models in several cases even when the training sets are very small.

Finally, we also note that our prioritization method is very fast, generally requiring, at most, only a few minutes for training. The most time-consuming parts are loading the data and computing the topic model, but these are actually performed only once when the project is first created.

### Document tagging

The tagging and annotation capabilities of SWIFT-Review can be useful during the activities of scoping and problem formulation. Together, they can be employed to more quickly assess the extent of available evidence, prioritize health outcomes and chemical exposures for systematic review, and understand the degree of evidence integration that may be required. In addition, the resulting visualizations and reports can help to identify topics that have been extensively studied as well as emerging areas of research. Topic modeling results can also be used to automatically uncover important themes found in a literature corpus and can help to identify “seed studies” for the purpose of training a machine-learning model that priority ranks relevant studies in focused areas.

Additional work is required to validate the accuracy of the tagging against manual review. We envision that refinements will be made to the current search filters used to classify health outcomes and evidence streams to improve accuracy based on results of validation work. However, we have presented a realistic case study for the use of SWIFT-Review for problem formulation and found that the tagging capabilities of SWIFT Review are useful to understand the relative data rich and data poor aspects of a topic of interest, for example the most studied health outcomes for a particular chemical or the relative proportion of evidence that is animal-based. Also, by interactively and iteratively exploring, tagging, and filtering the corpus, it is possible to use SWIFT-Review to efficiently enrich the corpus and bring promising research topics into clearer focus. Current practice at the NTP is to use the tagging features during problem formulation (or “scoping”) but to rely on manual tagging when implementing the formal systematic review. With respect to creating automatic search strategies for Tox21 and other chemicals, we find that automation greatly reduces the amount of time required to create draft search strategies (especially for topics that involve many chemicals) but that manual review of the automated search strategy is recommended. For example, by interactively and iteratively exploring, tagging, and filtering the corpus, it is possible to use SWIFT-Review to efficiently enrich the corpus and bring promising research topics into clearer focus.

### Limitations and future developments

One barrier for widespread uptake of priority-ranking methods like SWIFT-Review is the current inability to provide users with feedback on when to stop screening to achieve a desired percentage recall. This is an area we are actively investigating. Like related approaches [22–24], our method of detecting this stopping threshold may involve some amount of random sampling, a tactic which appears to work well, but will come at a cost in terms of WSS. Another barrier is that seed studies need to be identified to train the models, which can present an additional human screening burden. To address these issues, we and others [23, 25–27] are moving toward active learning and models that can be initialized without seed studies and then continuously updated during the screening process. Under this active learning framework, it also becomes more natural to implement sampling methods that can utilize feedback from the user in order to estimate at what point they can stop screening with confidence that a desired level of recall has been achieved for a particular data set. With some additional modifications, the prioritization method we have presented here can be modified to accommodate these improvements.

The public version of SWIFT-Review currently works with PubMed records only. Future developments will include the ability to upload non-PubMed records directly from an EndNote library, flat file, etc., as well as options to import full-text documents and enhancements to the automated tagging functions such as support for gene names, new chemical lists, MeSH-on-demand, etc. In addition, in order to extend health outcome tagging to documents originating from alternate bibliographic databases as well as abstracts that have not yet been indexed by MeSH, we are currently preparing search strategies that can be used to tag documents according to the following broad categories of health outcome: body weight/growth, cancer, cardiovascular, dermatological, developmental, endocrine, gastrointestinal, hematological, immunological, hepatic, renal, metabolic, musculoskeletal, neurological, sensory, reproductive, and respiratory. These features will appear in future updates of the software.

## Conclusions

Text-mining and machine learning programs such as SWIFT-Review can be valuable tools to reduce the human screening burden and assist in problem formulation. The freely available SWIFT-Review software is currently being used by researchers in government, academic, non-profit, and for-profit organizations and is under ongoing development, with several new features planned.

## Additional files

Additional file 1: OHAT datasets. (XLSX 4022 kb) Additional file 2: CAMARADES dataset. (XLSX 17704 kb) Additional file 3 Evidence stream search strategies. (DOCX 26 kb) Additional file 4: Tox21 chemical names and synonyms. (XLSX 3996 kb) Additional file 5: Exposure search strategies. (DOCX 27 kb) Additional file 6: UNEP EDCs. (XLSX 1432 kb) Additional file 7: High ranking classification terms. (DOCX 45 kb)

## Abbreviations

ADHD: attention deficit hyperactivity disorder
BPA: bisphenol-A
CAMARADES: Collaborative Approach to Meta-Analysis and Review of Animal Data from Experimental Studies
CASRN: Chemical Abstract Services Registry Number
EDC: endocrine-disrupting chemical
EPA: Environmental Protection Agency
FDA: Food and Drug Administration
FN: false negative
LBFGS: limited memory Broyden-Fletcher-Goldfarb-Shanno
LDA: latent Dirichlet allocation
MeSH: Medical Subject Heading
NCATS: National Center for Advancing Translational Sciences
NCBI: National Center for Biotechnology Information
NIEHS: National Institute of Environmental Health Sciences
NIH: National Institutes of Health
NTP: National Toxicology Program
OHAT: Office of Health Assessment and Translation
PAH: polycyclic aromatic hydrocarbon
PFOA: perfluorooctanoic Acid
PFOS: perfluoroocane sulfonate
PMID: PubMed ID
SWIFT: Sciome Workbench for Interactive computer-Facilitated Text-mining
TF-IDF: term frequency-inverse document frequency
TN: true negative
TP: true positive
UNEP: United Nations Environment Programme
WHO: World Health Organization
WSS: Work Saved over Sampling
