# Supplementary material for: SWIFT-Review: a text-mining workbench for systematic review
Source: Syst Rev. 2016 May 23;5:87. doi: 10.1186/s13643-016-0263-z (PMC4877757; doi:10.1186/s13643-016-0263-z)
Supplement: Additional file 3 — Evidence stream search strategies. (DOCX 26 kb) [file 13643_2016_263_MOESM3_ESM.docx]

# Evidence Stream Search Strategy

## Animal

mesh_mh: ("animal experimentation" OR "models, animal" OR "behavior, animal" OR "animal population groups" OR "invertebrates" OR "chordata, nonvertebrate" OR "amphibians" OR "birds" OR "fishes" OR "reptiles" OR "artiodactyla" OR "carnivora" OR "cetacea" OR "chiroptera" OR "elephants" OR "hyraxes" OR "insectivora" OR "lagomorpha" OR "marsupialia" OR "monotremata" OR "perissodactyla" OR "rodentia" OR "scandentia" OR "sirenia" OR "xenarthra" OR "strepsirhini" OR "platyrrhini" OR "tarsii" OR "cercopithecidae" OR "hylobatidae" OR "gorilla gorilla" OR "pan paniscus" OR "pan troglodytes" OR "pongo pygmaeus") OR

mesh_mh_noexp: ("Animals" OR "chordata" OR "vertebrates" OR "mammals" OR "primates" OR "haplorhini" OR "catarrhini" OR "hominidae") OR

tiab: (animal* OR mice OR mus OR mouse OR murine OR rats OR rat OR murinae OR muridae OR "cotton rat" OR "cotton rats" OR hamster* OR criceti?ae OR rodent* OR pigs OR pig OR swine* OR piglet* OR "guinea pigs" OR "guinea pig" OR cavia OR callithrix OR marmoset* OR cebuella OR hapale OR octodon OR chinchilla* OR gerbillinae OR gerbil* OR rabbit* OR hares OR hare OR cats OR cat OR carus OR felis OR dogs OR dog OR canine* OR canis OR sheep* OR mouflon* OR ovis OR goat* OR capra OR haplorhini OR monkey* OR anthropoid* OR saguinus OR tamarin* OR leontopithecus OR hominidae OR ape OR apes OR "pan paniscus" OR bonobo* OR "pan troglodytes" OR gibbon* OR nomascus OR symphalangus OR chimpanzee* OR prosimian* OR pongidae OR gorilla* OR "pongo pygmaeus" OR orangutan* OR lemur* OR lemuridae OR horse* OR equus OR cow OR calf OR bull OR chicken* OR gallus OR quail* OR bird OR birds OR poultry OR fowl OR fowls OR reptil* OR snake* OR lizard* OR turtle* OR amphibia* OR frog* OR xenopus OR bombina OR salientia OR toad* OR "epidalea calamita" OR salamander* OR fish OR fishes OR pisces OR catfish OR perch OR percidae OR perca OR trout OR char OR salmon OR salvelinus OR minnow* OR cyprinidae OR carp OR zebrafish OR "zebra fish" OR nematode* OR elegans OR diptera OR flies OR dipteral OR drosophil* OR squirrel* OR chipmunk* OR bear OR bears OR ursidae OR dolphin* OR porpoise* OR whale* OR cetacea)

## Human

mesh_mh:( humans OR human development ) OR tiab: (human* OR individual* OR person* OR people OR population*) OR

mesh_mh:( age groups) OR tiab: (pediatric* OR paediatric* OR baby OR babies OR newborn* OR infant* OR toddler* OR child* OR youth* OR youngster* OR adolescen* OR pubert* OR juvenile* OR tween* OR teen OR teens OR teenager*) OR (tiab:("in utero" OR prenat* OR perinat* OR neonat* OR postnat*) AND NOT tiab: (mice OR mouse OR rat OR rats)) OR

tiab:(preschool* OR "pre-school*" OR kindergarten* OR schoolchild* OR student*) OR

tiab:(adult* OR "middle age*" OR aged OR elder* OR "senior citizen*" OR seniors OR retiree* OR septuagenarian* OR octagenarian* OR sexagenarian* OR nonagenarian* OR centenarian*) OR

mesh_mh:(nuclear family) OR tiab:(famil* OR parent* OR father* OR mother* OR sibling* OR brother* OR sister* OR twin OR twins OR step-father* OR step-mother* OR step-daughter* OR step-son* OR aunt* OR uncle* OR niece* OR nephew* OR grandparent* OR grandfather* OR grand-father* OR grandmother* grand-mother* OR grandchild* OR granddaughter* OR grandson* OR spouse* OR partner* OR husband* OR wife OR wives OR guardian* OR caregiver* OR "care giver*") OR

mesh_mh:(men OR women) OR tiab:(men OR man OR boy OR boys OR boyhood OR women OR woman OR girl OR girls OR girlhood) OR

mesh_mh:("population groups" OR "vulnerable populations") OR tiab:("african american*" OR "asian american*" OR hispanic* OR latina* OR latino* OR "mexican american*" OR underserved OR disadvantaged) OR

mesh_mh:("epidemiologic studies" OR "double-blind method" OR "single-blind method") OR

mesh_sh:(epidemiology) OR tiab:("case control*" OR cohort OR "cross sectional" OR "follow-up study" OR longitudinal OR prospective OR retrospective) OR

mesh_pubtype:("case reports" OR "clinical trial" OR "observational study" OR "randomized control trial" OR "twin study") OR tiab:("clinical trial*" OR observational OR "randomized control trial*") OR

mesh_mh:("research subjects" OR "human experimentation" OR patients OR "Patient Participation") OR tiab:("human subject*" OR "research subject*" OR client* OR patient* OR inpatient* OR outpatient* OR participant* OR volunteer*) OR

mesh_mh:("occupational groups" OR "occupational exposure") OR tiab:(occupation* OR workplace OR "work place" OR "work-related" OR administrator* OR aides OR assistant* OR crew OR crews OR employee* OR personnel OR professional OR staff OR technician* OR worker* OR educator* OR instructor* OR teacher* OR clinician* OR doctor* OR physician* OR pharmacist* OR nurs* OR residents OR veterinarian*)

## In Vitro

mesh_mh:("Cell line" OR "tumor cells, cultured" OR "in vitro techniques") OR

tiab:"primary cells"~4 OR

tiab:("cell line" OR "cell lines" OR "cell culture" OR cultured OR "in vitro" OR 3T3 OR A549 OR "BEAS-2B" OR "CACO-2" OR "CHO cells" OR HELA OR HepG2 OR HepaRG OR Jurkat OR "MCF-7" OR "stem cells")
