# Supplementary material for: SWIFT-Review: a text-mining workbench for systematic review
Source: Syst Rev. 2016 May 23;5:87. doi: 10.1186/s13643-016-0263-z (PMC4877757; doi:10.1186/s13643-016-0263-z)
Supplement: Additional file 5: — Exposure search strategies. (DOCX 27 kb) [file 13643_2016_263_MOESM5_ESM.docx]

# Exposure Search Strategy

## Air Pollution

( mesh_mh:( "air pollution" OR "air pollutants" OR "particulate matter" OR smog OR soot OR "vehicle emissions" OR "motor vehicles" )) OR

pharm_actions:"air pollutants" OR

( tiab: ( "air pollution" OR "air pollutant" OR "air pollutants" OR "particulate matter" OR "PM2.5" OR "PM(2.5)" OR PM10 OR "PM(10)" OR smog OR soot OR "carbon black" OR "black carbon" OR "elemental carbon" )) OR

( tiab:(( air OR airborne OR coarse OR ultrafine OR fine ) AND ( particle* OR particulate*))) OR

( tiab:(( vehicle OR vehicles OR vehicular OR auto OR automobile OR bus OR buses OR car OR truck* OR engine OR traffic OR transport* ) AND ( emissions OR exhaust OR fume* ))) OR

(( tiab:( air OR outdoor* OR outside OR ambient OR pollut* OR emissions OR exhaust* )) AND

((( tiab:( S02 OR "sulfur dioxide" OR ozone OR O3 OR "hydrogen sulfide" OR H2S OR "carbon monoxide" OR "nitric oxide" OR "nitrogen oxide" OR "nitrogen oxides" OR "nitrogen dioxide" OR "NOx" OR "NO(x)" OR NO2 ))) OR

( mesh_mh:( "sulfur dioxide" OR ozone OR "hydrogen sulfide" OR "carbon monoxide" OR "nitrogen dioxide" ))) OR

mesh_mh:( "volatile organic compounds" OR "fossil fuels" ) OR

tiab:( "volatile organic compounds" OR gasoline* OR diesel OR petrol* ) OR

mesh_mh_noexp:( "Polycyclic hydrocarbons, aromatic" ) OR

mesh_mh:( "benzo(a)pyrene" OR benzene ) OR

tiab:( "polycyclic aromatic hydrocarbon*" OR "benzopyrene" OR "benzo-a-pyrene" OR "3,4-benzopyrene" OR benzene )) OR

tiab:( indoors OR "air quality" ) OR

tiab:( indoor AND ( "air pollution" OR smoke )) OR

mesh_mh:( "tobacco smoke pollution" OR smoking ) OR

tiab:( "secondhand smoke" OR "secondhand smoking" OR "second hand smoke" OR "second hand smoking" OR "passive smoke" OR "passive smoking" ) OR

tiab:(( smoke OR smoking) AND (cigarette OR tobacco OR cigar*)) OR

tiab:woodsmoke OR

(( mesh_mh:wood OR

tiab:( wood OR firewood OR biomass* OR charcoal OR fuel OR fuels OR gas OR gasoline OR kerosene OR dung OR manure )) AND

( mesh_mh:smoke OR

tiab:( smoke OR smoking OR combust* OR burn* OR burning ))) OR

mesh_mh:radon OR tiab:radon

## Allergens

mesh_mh:( allergens OR dust ) OR tiab:( allerg* )

## Diet and Nutrition

mesh_mh:( diet OR "dietary supplements" OR "maternal nutritional physiological phenomena" OR "child nutritional physiological phenomena") OR

tiab:( diet OR diets OR dietary OR "dietary supplement*" OR fasting OR nutrition* OR nutrient* OR micronutrient*) OR

tiab:(( maternal OR mother* OR paternal OR father*) AND ( diet OR dietary OR nutrition)) OR

tiab:(( caloric OR calorie* OR food OR nutrient* OR nutrition*) AND ( excess OR intake* OR restrict* )) OR

mesh_mh:( Isoflavones OR genistein ) OR

tiab:( isoflavone* OR isoflavonoid* OR genistein OR soy OR soybean* OR "soy beans" OR phytoestrogen* OR phytochemical* ) OR

tiab:( "food additive*" OR "folic acid" OR folate OR "high fat diet*" OR "low protein*" OR "caloric restrict*" OR "calorie restric*" )

## Drugs of Abuse

mesh_mh:( "street drugs" OR "cocaine" OR "amphetamines" OR "caffeine" OR "nicotine" OR "tobacco" OR "smoking" OR "tobacco smoke pollution" OR morphine OR cannabis ) OR

pharm_actions:( narcotics ) OR

tiab:((drug OR drugs) AND (addict* OR abuse OR illicit OR recreational OR street)) OR

tiab:( "controlled substances" OR "substance abuse" OR

cocaine OR amphetamine* OR methamphetamine* OR methylamphetamine* OR metamfetamine* OR caffein* OR nicotine OR tobacco OR smoking OR cigarette* OR morphine OR dihydromorphine OR hydromorphone OR oxymorphone OR phencyclidine OR codeine OR heroin OR heroine OR thebaine OR cannabis OR marijuana OR buprenorphine OR methadone OR opium OR opiate* OR oxycodone OR tramadol) OR

mesh_mh:( "alcohol-related disorders" OR ethanol OR "alcoholic beverages" ) OR

tiab:( alcohol* OR liquor* OR beer OR wine OR spirits )

## Endocrine Disruptors

mesh_mh:( "endocrine disruptors" OR "diethylstilbestrol" ) OR

tiab:( diethylstilbestrol OR stilbestrol OR "stilbene estrogen" OR xenoestrogen* ) OR

tiab:( endocrine AND disrupt* )

## Flame Retardants

mesh_mh:( "flame retardants" OR "halogenated diphenyl ethers" ) OR

pharm_actions:( "flame retardants" ) OR

tiab:( PBDE* OR PCDE* ) OR

tiab:((fire OR flame) AND retardant*) OR

tiab:((halogenated OR chlorinated OR brominated OR polybrominated) AND "diphenyl ethers")

## General Environmental Exposure

(mesh_mh:( "specialty uses of chemicals" OR "toxic actions" )) OR

( mesh_mh_noexp:(environment OR "environmental pollutants" OR "noxae" OR "environmental pollution" )) OR

( mesh_mh:( "air pollutants" OR "carcinogens, environmental" OR "endocrine disruptors" OR "hazardous substances" OR "water pollutants" OR "carcinogens" OR "cardiotoxins" OR "cytotoxins" OR "dermotoxins" OR "immunotoxins" OR "mutagens" OR "neurotoxins" OR "teratogens" OR "pesticides" OR "air pollution" OR "environmental exposure" OR "water pollution" )) OR

( pharm_actions:("hazardous substances" OR "environmental pollutants" )) OR

( tiab:( xenobiotic* OR xenoestrogen* )) OR

(tiab:( "environmental agent*" OR "environmental chemical*" OR "environmental compound*" OR "environmental contaminant*" OR "environmental determinant*" OR "environmental estrogen*" OR "environmental exposure*" OR "environmental factor*" OR "environmental influence*" OR "environmental stress*" OR "environmental epigenetic*" )) OR

( tiab:( carcinogen OR carcinogens OR carcinogenic OR teratogen OR teratogenic OR mutagen OR mutagens OR mutagenic OR pollutant* OR pollution OR cardiotox* OR dermotox* OR immunotox* OR nephrotox* OR neurotox* OR toxicant* OR toxin* ))

OR ( mesh_mh:teratogens ) OR

( title:( environment* AND epigen*)) OR

( tiab:( "chemical compound*" OR "chemical exposure*" OR "chemical mixture*" OR "chemical product*" OR "chemical substance*" OR "hazardous compound*" OR "hazardous exposure*" OR "hazardous mixture*" OR "hazardous product*" OR "hazardous substance*" OR "industrial compound*" OR "industrial chemical*" ))

## Heavy Metals

mesh_mh:"metals, heavy" OR

tiab:( "heavy metal" OR "heavy metals" ) OR

(( mesh_mh:( arsenic OR arsenicals OR "lead poisoning" OR "methylmercury compounds" ) OR

tiab:( metals OR arsenic OR arsenical* OR arsenite* OR arsenate* OR cadmium OR chromium OR cobalt OR

(( metal OR metals) AND lead ) OR

mercury OR nickel OR tin OR vanadium OR methylmercury )) AND

( tiab:( exposed OR exposure* OR induce* OR toxic OR toxicity OR toxin* )))

## Ionizing Radiation

mesh_mh:( "x-rays" ) OR

tiab:( "x-ray*" OR radiation OR irradiated )

## Miscellaneous

mesh_mh:( formaldehyde OR organotin OR nitrates ) OR

tiab:( "1,3-butadiene" OR formaldehyde OR organotin* OR tributyltin OR nitrate* )

## Occupational

( mesh_mh:"occupational exposure" ) OR

( tiab: (( occupation* OR workplace OR "work place" OR "work related" OR worker* OR employee* ) AND

( expos* OR chemical* OR hazard*)))

## Pesticides

mesh_mh:pesticides OR

pharm_actions:pesticides OR

tiab:( pesticid* OR fungicide* OR herbicide* OR insecticide* OR repellent* OR rodenticide* ) OR

mesh_mh:( "hydrocarbons chlorinated" ) OR

tiab:( organochlorin* OR "organic chlorine" OR "chlorinated hydrocarbons" OR aldrin OR chlordan OR chlorobenzene* OR chlorofluorocarbon* OR "dichlorophenyl dichloroethylene" OR dichlorodiphenyltrichloroethane* OR dieldrin OR endrin OR heptachlor OR methoxychlor OR "polychlorinated biphenyl" OR "polychlorinated biphenyls" OR polychlorobiphenyl OR PCBs OR tetrachloroethylene OR trichloroethane* OR "vinyl chloride" ) OR

tiab:( "agent orange" OR Amitraz OR atrazine OR avermectin OR captan OR carbaryl carbofuran OR chlorfenvinphos OR chlorpyrifos OR coumaphos OR DDT OR deet OR "N,N-diethyltoluamide" OR diazinon OR dichlorvos OR "dimethyl phthalate" OR endosulfan OR heptachlor OR linalool OR malathion OR paraquat OR parathion OR pentachlorophenol OR permethrin OR pyrethrin* OR rotenone OR vinclozolin OR warfarin ) OR

mesh_mh:dioxins OR

tiab:( dioxin* OR dioxane* OR "2,3,7,8-tetrachlorodibenzo-p-dioxin" OR tetrachlorodibenzodioxin OR TCDD )

## Phthalates

mesh_mh:( Plasticizers OR "dibutyl phthalate" ) OR

pharm_actions:"plasticizers" OR

tiab:( phthalate* OR "dibutyl phthalate" OR "butyl phthalate" OR "butyl benzyl phthalate" OR "Bisphenol A" )

## Polycyclic Aromatic Hydrocarbons

mesh_mh_noexp:( "hydrocarbons, aromatic" OR "polycyclic hydrocarbons, aromatic" ) OR

tiab:( "aromatic hydrocarbons" OR "polycyclic aromatic hydrocarbons" OR PAHs ) OR

mesh_mh:( "benzo(a)pyrene" OR xylenes OR benzene ) OR

tiab:( "benzopyrene" OR "benzo a pyrene" OR "3,4-benzopyrene" OR benzene OR toluene OR xylene )

## Solvents

mesh_mh:solvents OR

tiab:( solvents OR "organic solvent" OR "2-bromopropane" OR "2-propanol" OR acetone OR tetrachloroethylene OR toluene OR trichloroethylene )

## Stress

mesh_mh:"stress, psychological" OR

(( title:stress OR tiab:stressed) AND NOT ( tiab:( "oxidatives tress" OR "osmotic stress" OR "redox stress" ))) OR

mesh_mh:( grooming OR "maternal behavior" ) OR

tiab:( "maternal care"~3 OR "maternal behav*"~3 OR "maternal groom*"~3 )

tiab:( "maternal adversity" OR "prenatal stress*" OR "emotional state*" OR "distress" OR mood OR anxiety OR anxious OR fear )
