# Supplementary material for: SWIFT-Review: a text-mining workbench for systematic review
Source: Syst Rev. 2016 May 23;5:87. doi: 10.1186/s13643-016-0263-z (PMC4877757; doi:10.1186/s13643-016-0263-z)
Supplement: Additional file 7: — High ranking classification terms. (DOCX 45 kb) [file 13643_2016_263_MOESM7_ESM.docx]

Tables 1-4 list the most highly weighted features (positive weights in green, negative in red) in the resulting models for each of the OHAT datasets (trained using *N*=100).

**Table 1:** Examples of High Ranking Features, BPA Dataset (No MeSH)

| Label | Feature name | Abs(Weight) | |
| --- | --- | --- | --- |
| Included | Topic 87: effects, environmental, exposure, health, human, studies, chemicals, bisphenol, potential, endocrine | | 0.719875 |
| Included | Topic 12: bpa, urinary, bisphenol, association, levels, exposure, women, concentrations, age, higher | | 0.504291 |
| Included | Topic 83: exposure, bpa, mammary, fetal, development, offspring, prenatal, exposed, maternal, increased | | 0.44122 |
| Included | Topic 88: ppargamma, activation, expression, peroxisome, proliferator-activated, badge, receptor, rosiglitazone, cells, ppar-gamma | | 0.401426 |
| Included | Abstract_obes | | 0.34092 |
| Included | Title_obes | | 0.325999 |
| Included | Title_endocrin | | 0.272045 |
| Included | Title_children | | 0.264878 |
| Included | Topic 4: receptor, bpa, bisphenol, binding, receptors, human, estrogen, nuclear, activity, er? | | 0.230883 |
| Included | Title_adipos | | 0.228953 |
| Included | Topic 84: endocrine, edcs, chemicals, disrupting, compounds, disruptors, nonylphenol, endocrine-disrupting, environmental, edc | | 0.227858 |
| Included | Title_birth | | 0.21469 |
| Included | Abstract_adiponectin | | 0.212202 |
| Included | Abstract_bpa | | 0.21055 |
| Included | Abstract_adipos | | 0.197756 |
| Included | Title_disrupt | | 0.196572 |
| Included | Abstract_adipocyt | | 0.191678 |
| Included | Title_adipocyt | | 0.189216 |
| Included | Title_human | | 0.185294 |
| Included | Abstract_weight | | 0.183488 |
| Included | Abstract_diet | | 0.182933 |
| Included | Abstract_receptor | | 0.182071 |
| Included | Abstract_metabol | | 0.177073 |
| Included | Abstract_leptin | | 0.175578 |
| Included | Title 2-Gram_and obes | | 0.174485 |
| Included | Title 3-Gram_endocrin disrupt chemic | | 0.17352 |
| Included | Title_disruptor | | 0.17173 |
| Included | Title_express | | 0.166253 |
| Excluded | Topic 34: study, results, high, found, compared, analysis, time, present, showed, observed | | 0.511471 |
| Excluded | Topic 66: epoxy, contact, resin, allergic, dermatitis, bisphenol, resins, occupational, patients, sensitization | | 0.300503 |
| Excluded | Title_dental | | 0.296741 |
| Excluded | Topic 37: pulp, materials, filling, tissue, healing, root, pulpal, days, chamber, calcium | | 0.285053 |
| Excluded | Title_materi | | 0.273902 |
| Excluded | Topic 64: restorations, clinical, composite, evaluation, resin, years, year, marginal, class, months | | 0.266332 |
| Excluded | Topic 72: materials, dental, material, clinical, composite, applications, restorative, technique, cortoss, techniques | | 0.257628 |
| Excluded | Topic 7: water, river, concentrations, samples, surface, compounds, bisphenol, sediment, waters, nonylphenol | | 0.237844 |
| Excluded | Topic 24: bpa, degradation, bisphenol, reaction, oxidation, presence, water, concentration, aqueous, solution | | 0.232287 |
| Excluded | Title_light | | 0.216568 |
| Excluded | Title_us | | 0.204472 |
| Excluded | Topic 50: sealant, sealants, fissure, pit, caries, occlusal, dental, sealing, sealed, placement | | 0.194121 |
| Excluded | Title_microleakag | | 0.185905 |
| Excluded | Title_composit | | 0.184198 |
| Excluded | Title 3-Gram_materi and estrogen | | 0.179927 |
| Excluded | Topic 55: microleakage, composite, restorations, class, groups, teeth, margins, marginal, leakage, resin | | 0.179799 |
| Excluded | Topic 58: bonding, adhesive, dentin, bond, systems, adhesives, self-etching, single, agents, prime | | 0.176121 |
| Excluded | Topic 73: orthodontic, brackets, bonded, adhesive, bonding, transbond, bond, adhesives, teeth, groups | | 0.174784 |
| Excluded | Title_from | | 0.170329 |
| Excluded | Title_sealant | | 0.167521 |
| Excluded | Abstract_bi | | 0.165819 |
| Excluded | Topic 22: dental, monomers, composite, resin, tegdma, bisgma, components, bis-gma, composites, dimethacrylate | | 0.165804 |

**Table 2:** Examples of High Ranking Features, PFOS/PFOA Dataset (No MeSH)

| Label | | Feature name | Abs(Weight) |
| --- | --- | --- | --- |
| Included | Topic 97: mice, pfoa, activation, alpha, pparalpha, peroxisome, receptor, mouse, expression, ppar? | 0.585887 |  |
| Included | Topic 27: pfoa, serum, levels, exposure, acid, association, study, health, perfluorooctanoic, associations | 0.427131 |  |
| Included | Topic 70: rats, pfoa, mice, mg/kg, exposure, dose, day, weight, days, female | 0.42269 |  |
| Included | Topic 48: liver, serum, rats, hepatic, levels, ammonium, perfluorooctanoate, increased, ppm, testosterone | 0.384877 |  |
| Included | Abstract_pfo | 0.318412 |  |
| Included | Abstract_pfoa | 0.303064 |  |
| Included | Title_mice | 0.278569 |  |
| Included | Abstract_mice | 0.26988 |  |
| Included | Abstract_il | 0.26751 |  |
| Included | Abstract_exposur | 0.255134 |  |
| Included | Abstract_serum | 0.238704 |  |
| Included | Abstract_immun | 0.23774 |  |
| Included | Title_alter | 0.234146 |  |
| Included | Topic 5: expression, genes, gene, exposure, mrna, cyp, protein, levels, thyroid, acid | 0.230875 |  |
| Included | Topic 72: pfos, perfluorooctane, sulfonate, exposure, effects, exposed, concentrations, study, toxicity, zebrafish | 0.228099 |  |
| Included | Title_perfluorooctano | 0.211267 |  |
| Included | Topic 49: acid, pfoa, liver, peroxisome, perfluorooctanoic, fatty, rats, activity, hepatic, peroxisomal | 0.209407 |  |
| Included | Abstract_dai | 0.193732 |  |
| Included | Title_perfluorooctanesulfon | 0.193111 |  |
| Included | Abstract_dose | 0.192394 |  |
| Included | Title_exposur | 0.191278 |  |
| Included | Title_pfo | 0.1894 |  |
| Included | Abstract_mg | 0.189155 |  |
| Included | Abstract_ig | 0.183927 |  |
| Included | Title_immun | 0.178981 |  |
| Included | Title_cytokin | 0.171091 |  |
| Included | Topic 71: maternal, cord, birth, exposure, fetal, weight, blood, pregnant, perfluorinated, women | 0.168164 |  |
| Included | Abstract_pp | 0.165828 |  |
| Excluded | Topic 14: study, results, observed, compared, found, time, data, high, showed, significant | 0.43317 |  |
| Excluded | Topic 21: high, application, potential, applications, studies, development, low, results, approach, developed | 0.337509 |  |
| Excluded | Title_oxygen | 0.280311 |  |
| Excluded | Topic 59: acids, pfcas, perfluorinated, chain, perfluoroalkyl, isomers, carboxylic, pfca, branched, pfas | 0.252301 |  |
| Excluded | Topic 36: perfusion, hearts, heart, ischemia, degrees, myocardial, perfluorochemical, reperfusion, cardiac, group | 0.245048 |  |
| Excluded | Topic 24: lung, ventilation, plv, partial, liquid, injury, pulmonary, gas, exchange, acute | 0.234834 |  |
| Excluded | Topic 10: concentrations, trends, arctic, temporal, samples, pfcs, sea, compounds, perfluoroalkyl, pfcas | 0.228505 |  |
| Excluded | Title_liquid | 0.22616 |  |
| Excluded | Topic 90: oil, silicone, vitreous, perfluorocarbon, liquids, perfluorodecalin, eyes, retinal, liquid, retina | 0.213681 |  |
| Excluded | Topic 64: fluorinated, polymer, polymers, properties, materials, thermal, low, temperature, high, fluorine | 0.210205 |  |
| Excluded | Topic 20: stent, muscle, everolimus-eluting, trial, tests, biocompatibility, stents, tissue, hematopoietic, wound | 0.202028 |  |
| Excluded | Topic 60: blood, oxygen, substitutes, carriers, artificial, clinical, hemoglobin, red, transfusion, cell | 0.197896 |  |
| Excluded | Topic 86: oxygen, emulsions, pfc, perfluorocarbon, perfluorocarbons, pfcs, pfob, emulsion, transport, size | 0.188629 |  |
| Excluded | Abstract_chain | 0.186294 |  |
| Excluded | Topic 87: surface, fluorinated, polymer, block, polymers, molecular, copolymers, chains, copolymer, spectroscopy | 0.184208 |  |
| Excluded | Abstract_pce | 0.17843 |  |
| Excluded | Abstract_surfac | 0.177028 |  |
| Excluded | Title_perfluorocarbon | 0.176478 |  |
| Excluded | Topic 56: pfoa, acid, degradation, decomposition, reaction, oxidation, atmospheric, water, rate, photolysis | 0.171973 |  |
| Excluded | Abstract_calcium | 0.169774 |  |
| Excluded | Title_albumin | 0.169586 |  |
| Excluded | Title_make | 0.166867 |  |

**Table 3:** Examples of High Ranking Features, Transgenerational Dataset (No MeSH)

| Label | Feature name | Abs(Weight) |
| --- | --- | --- |
| Included | Topic 43: effects, development, environmental, role, studies, mechanisms, factors, evidence, human, life | 0.688915 |
| Included | Topic 86: rats, reproductive, effects, offspring, female, study, exposure, male, generation, females | 0.627736905 |
| Included | Topic 54: diet, acid, rats, fatty, fed, acids, dietary, oil, diets, content | 0.608731727 |
| Included | Title_epigenet | 0.412205023 |
| Included | Title_transgener | 0.411989777 |
| Included | Topic 85: exposure, radiation, sperm, damage, effects, exposed, dna, irradiation, instability, mutations | 0.396286068 |
| Included | Topic 34: epigenetic, dna, methylation, gene, genes, expression, transgenerational, germ, inheritance, generations | 0.360939885 |
| Included | Topic 9: effects, exposure, generations, eggs, development, survival, generation, exposed, concentrations, reproduction | 0.349051565 |
| Included | Topic 56: disorder, disorders, depression, cognitive, stress, anxiety, symptoms, psychiatric, memory, behavioral | 0.326407395 |
| Included | Abstract_epigenet | 0.281180413 |
| Included | Abstract_rat | 0.249490471 |
| Included | Topic 69: maternal, birth, mothers, pregnancy, offspring, women, infants, infant, fetal, age | 0.240847329 |
| Included | Title_effect | 0.230804967 |
| Included | Title_rat | 0.229031943 |
| Included | Title_environ | 0.222374128 |
| Included | Topic 93: immigrants, ethnic, population, generation, american, groups, immigrant, health, asian, social | 0.21043566 |
| Included | Abstract_edc | 0.207987849 |
| Included | Abstract_offspr | 0.203837828 |
| Included | Title_diet | 0.197952135 |
| Included | Title 3-Gram_transgener epigenet inherit | 0.181107152 |
| Included | Title_pattern | 0.165368405 |
| Excluded | Topic 87: system, x-ray, radiation, synchrotron, resolution, imaging, image, laser, third-generation, light | 0.435001282 |
| Excluded | Topic 68: family, syndrome, generations, dominant, autosomal, familial, affected, members, congenital, inheritance | 0.292664949 |
| Excluded | Title 3-Gram_third gener pill | 0.26640688 |
| Excluded | Topic 44: treatment, clinical, therapy, patients, agents, efficacy, trials, effective, drugs, therapeutic | 0.249872986 |
| Excluded | Title 2-Gram_gener pill | 0.244392731 |
| Excluded | Title_pill | 0.22968437 |
| Excluded | Topic 27: rats, hpv, blood, pressure, cervical, hypertensive, shr, rat, male, female | 0.226790901 |
| Excluded | Title_schizophrenia | 0.222196222 |
| Excluded | Topic 46: linkage, chromosome, markers, locus, genetic, gene, analysis, loci, region, map | 0.202418979 |
| Excluded | Topic 72: schizophrenia, antipsychotics, antipsychotic, treatment, second-generation, olanzapine, patients, risperidone, effects, disorder | 0.20123555 |
| Excluded | Title_tourett | 0.200527178 |
| Excluded | Topic 91: retinal, visual, eye, corneal, eyes, dystrophy, cataract, ocular, glaucoma, lens | 0.19508883 |
| Excluded | Title_thyrotropin | 0.194265668 |
| Excluded | Topic 12: infections, tract, urinary, treatment, respiratory, antibiotic, ceftriaxone, oral, cephalosporin, antibiotics | 0.189847764 |
| Excluded | Title_latent | 0.186957293 |
| Excluded | Topic 57: family, families, genetic, familial, disease, affected, members, generations, relatives, age | 0.186911802 |
| Excluded | Topic 26: oral, contraceptives, risk, women, venous, contraceptive, ocs, generation, thrombosis, third-generation | 0.183303432 |
| Excluded | Topic 42: repeat, mitochondrial, repeats, expansion, anticipation, cag, disease, mtdna, sca, instability | 0.176160881 |
| Excluded | Abstract_patient | 0.174998869 |
| Excluded | Abstract_gulo | 0.173535688 |
| Excluded | Topic 61: family, clinical, disease, dominant, generations, autosomal, affected, muscle, onset, familial | 0.169153992 |
| Excluded | Topic 20: inhibitors, compounds, activity, inhibitor, potent, drug, generation, second-generation, inhibition, drugs | 0.168165698 |
| Excluded | Topic 90: hepatitis, hcv, patients, virus, infection, anti-hcv, positive, liver, blood, antibodies | 0.165406151 |
| Excluded | Topic 45: chromosome, syndrome, translocation, normal, family, deletion, x-linked, generations, maternal, mother | 0.163886632 |
| Excluded | Abstract_cnvr | 0.162940535 |
| Excluded | Abstract_tourett | 0.162789493 |
| Excluded | Abstract_trab | 0.162636369 |
| Excluded | Abstract_mtdna | 0.161677698 |
| Excluded | Topic 14: gene, cells, tumor, mice, vectors, human, vector, virus, vivo, tumors | 0.161580138 |

**Table 4:** Examples of High Ranking Features, Neuropathic PainDataset (No MeSH)

| Label | Feature name | Abs(Weight) |
| --- | --- | --- |
| Included | Topic 0: nerve, neuropathic, injury, pain, spinal, ligation, peripheral, rats, sciatic, allodynia | 0.479143 |
| Included | Topic 52: nerve, cci, injury, sciatic, neuropathic, rats, chronic, constriction, pain, model | 0.448684 |
| Included | Topic 5: mechanical, allodynia, hyperalgesia, pain, thermal, rats, model, withdrawal, hypersensitivity, paw | 0.404257 |
| Included | Title_neuropath | 0.295667 |
| Included | Topic 44: pain, neuropathic, chronic, models, treatment, acute, mechanisms, model, elsevier, animal | 0.281713 |
| Included | Abstract_cci | 0.273325 |
| Included | Title_nerv | 0.265309 |
| Included | Title_injuri | 0.243473 |
| Included | Title_model | 0.236312 |
| Included | Abstract_nerv | 0.233496 |
| Included | Topic 60: diabetic, neuropathy, diabetes, rats, nerve, peripheral, conduction, treatment, weeks, streptozotocin | 0.222773 |
| Included | Topic 88: spinal, cord, dorsal, horn, expression, neurons, rat, c-fos, laminae, increased | 0.219537 |
| Included | Title_chronic | 0.215224 |
| Included | Abstract_neuropath | 0.211611 |
| Included | Title_pain | 0.201592 |
| Included | Title_allodynia | 0.191297 |
| Included | Title_hyperalgesia | 0.189667 |
| Included | Abstract_msh | 0.180727 |
| Included | Title 2-Gram_neuropath pain | 0.180289 |
| Included | Title_snl | 0.178032 |
| Included | Abstract_sciatic | 0.171488 |
| Included | Abstract_acrylamid | 0.171477 |
| Included | Title 2-Gram_on the | 0.160989 |
| Included | Abstract_snl | 0.157462 |
| Included | Title_selol | 0.156925 |
| Included | Abstract_selol | 0.154095 |
| Excluded | Topic 7: diabetic, rats, diabetes, vascular, responses, response, relaxation, control, arteries, muscle | 0.292018 |
| Excluded | Topic 91: hyperalgesia, injection, paw, thermal, nociceptive, formalin, inflammatory, rats, induced, rat | 0.26056 |
| Excluded | Topic 90: clinical, therapeutic, disease, studies, review, development, potential, models, animal, recent | 0.237113 |
| Excluded | Title_role | 0.233859 |
| Excluded | Title_agonist | 0.227598 |
| Excluded | Topic 77: treatment, patients, clinical, therapy, studies, management, trials, review, efficacy, effective | 0.224898 |
| Excluded | Topic 93: model, studies, study, animal, data, time, results, analysis, models, human | 0.224662 |
| Excluded | Topic 32: neurons, currents, current, drg, ganglion, dorsal, root, excitability, rat, sensory | 0.222851 |
| Excluded | Topic 95: case, patient, diagnosis, cases, pain, report, syndrome, disease, symptoms, revealed | 0.215847 |
| Excluded | Title_repair | 0.199155 |
| Excluded | Topic 72: synaptic, spinal, neurons, dorsal, horn, transmission, inhibitory, cord, postsynaptic, excitatory | 0.191145 |
| Excluded | Title 3-Gram_injuri and repair | 0.182696 |
| Excluded | Abstract_bcp | 0.177821 |
| Excluded | Title 2-Gram_and repair | 0.174141 |
| Excluded | Abstract_remsd | 0.171937 |
| Excluded | Title_inhibitor | 0.16216 |
| Excluded | Title_test | 0.160896 |
| Excluded | Abstract_ten | 0.159778 |
| Excluded | Abstract_acid | 0.159102 |
| Excluded | Title_glutam | 0.15674 |
| Excluded | Abstract_inhibitor | 0.154414 |
| Excluded | Title_acid | 0.154387 |
| Excluded | Title 2-Gram_natur cb2 | 0.152172 |
| Excluded | Topic 14: inflammation, inflammatory, joint, arthritis, adjuvant, cfa, complete, freund's, injection, pain | 0.152098 |
